# Reliability and Accuracy of Standard Reference Procedures for Measurements of Trunk and Arm Postures in Ergonomics

**DOI:** 10.3390/bioengineering12010050

**Published:** 2025-01-09

**Authors:** Carl M. Lind, Ida-Märta Rhen, Mikael Forsman

**Affiliations:** 1 Unit of Occupational Medicine, Institute of Environmental Medicine, Karolinska Institutet, 171 77 Stockholm, Sweden; 2 Centre for Occupational and Environmental Medicine, Stockholm County Council, 113 65 Stockholm, Sweden; 3 Department of Industrial and Materials Science, Chalmers University of Technology, 412 96 Gothenburg, Sweden; 4 School of Engineering Sciences in Chemistry, Biotechnology and Health, KTH Royal Institute of Technology, 141 57 Stockholm, Sweden

**Keywords:** measurement error, measurement bias, work postures, calibration procedures, posture assessment, standard posture, plumb line test, normal standing position, inclinometry, wearables

## Abstract

Adequate reference procedures for obtaining the reference zero-angle position are important for precise and accurate posture measurements, but few studies have systematically investigated these. A limited number of previous studies suggest differences in accuracy between procedures, with some causing an underestimation of the true arm elevation angle when sensors are taped to the skin. The reliability of commonly used reference procedures for the measurement of the trunk posture is also not well explored, and alternative procedures may improve precision. Based on this identified gap, this study evaluated the test–retest reliability of the N-position (I-pose), i.e., the standard procedure for recording trunk postures, and compared it with two new alternative procedures. Additionally, the accuracy of the N-position for measuring arm elevation angles was compared with one alternative procedure. A total of 40 participants (22 women and 18 men) aged 26–70 years performed the reference procedures in a laboratory setting. Postures were recorded using a smart workwear system equipped with two inertial measurement units (IMUs) embedded in pockets within the workwear. For the trunk posture, the N-position showed a slight lack of test–retest reliability, while one of the alternative procedures demonstrated better test–retest reliability. For the arm posture, the N-position, which does not include lateral trunk inclination, resulted in a substantial underestimation of the arm elevation angle of approximately 15°, which is a novel finding. In contrast, the posture involving trunk inclination closely matched the targeted reference, with a difference of less than 2°. This study underscores the importance of selecting appropriate reference procedures to ensure precise and accurate posture measurements.

## 1. Introduction

Musculoskeletal disorders (MSDs) of the lower back and neck are significant global health concerns, accounting for approximately 95 million disability-adjusted life years (DALYs) annually, while other musculoskeletal disorders collectively account for over 33 million DALYs worldwide [1]. Shoulder pain is prevalent globally with a seven-day prevalence of up to approximately 34% and a 12-month prevalence of up to around 55% [2].

The economic burden of work-related musculoskeletal disorders (WMSDs) and occupational accidents and diseases has been estimated at 3.3% of the gross domestic product in the European Union and 3.9% of the global gross domestic product [3]. WMSDs contribute to increased risks of short- and long-term work absenteeism, a reduced work capacity, and a premature exit from the labor market [4,5,6]. WMSDs are multifactorial, with both physical and psychosocial risk factors playing a role [7,8,9]. Important physical risk factors include strenuous postures and movements [7,10,11,12,13,14,15,16], strenuous and repetitive manual handling [7,10,12,16,17,18,19,20,21,22], and whole-body and hand–arm vibrations [7,10,23,24]. Accurate and precise measurements of these risk factors are important for establishing exposure–effect relationships and for risk assessment [25,26,27,28,29].

### 1.1. Sensor-Based Motion Capture Devices in Risk Prevention

Recent advancements in microelectronics have facilitated the increased use of sensor-based measurement instruments, such as triaxial accelerometers and inertial measurement units (IMUs), in research and occupational health practice. These tools enable more accurate assessments of work postures [27], complementing the traditional assessment that primarily relies on observation-based tools [25,26,30,31,32], enabling extended measurement durations from a few minutes to several hours or full workdays, providing more representative exposure measurements.

To increase the ease of use, motion devices can be integrated into workwear and connected to systems that enable the automated analysis and real-time monitoring of workers [27,33,34]. Examples of such sensor-based solutions include stretchable textile sensors attached on the outer surface of the workwear [35,36] and IMUs placed in embedded pockets in the workwear [37,38,39,40]. Stretchable textile sensors can be used to distinguish gross physical activity types [35,36], but generally exhibit lower accuracy compared to IMUs embedded in workwear. Embedding motion devices such as IMUs into workwear eliminates the need for skin attachment using tape or straps, which sometimes can be inconvenient and sensitive, as it may require partial undressing, access to private spaces, and anatomical knowledge for precise sensor placement.

### 1.2. Review of Reference Procedures for Accurate and Reliable Sensor-Based Measurements of Sagittal Trunk Inclination and Arm Elevation

In addition to accurate instrumentation, precise reference postures (i.e., procedures for obtaining the reference zero-angle position, typically a neutral body posture) are essential for obtaining accurate and reliable measurements and to make studies inter-comparable. While several studies have assessed, e.g., the reliability and inter-rater reliability of instruments and methods to assess the range of motion, such as for lumbar flexion [41,42,43], and the accuracy of inertia-based wearable motion capture systems against marker-based motion capture systems [44,45,46,47], less attention has been given to evaluating the reliability of reference positions for a neutral position for the trunk and upper arm. Ideally, a reference procedure (i.e., reference positions or postures and other arrangements such as instructions, assistance, and the use of assisting objects like chairs and external loads) should not only provide accurate and reliable measurements but also be practical for use in both laboratory and field studies.

To record the neutral posture for the trunk, arms, and head (or neck), Aarås and Stranden [48] proposed an upright, well-balanced position where the shoulders are relaxed and the upper arms hang naturally alongside the body without holding an external load. This reference procedure allows for simultaneous zero-posture recordings of the trunk, head, and arms.

The posture is often referred to as the natural, normal, or standard posture, the normal stance [49,50], or the N-position, N-posture, or I-pose [51] (hereafter to be referred to as the N-position). Variations of the N-position have been widely used in ergonomics research to establish the reference zero-angle position for both the trunk (see Table 1) and the upper arm (see Table 2).

As shown in Table 1, the N-position appears to be the most used zero posture. Among the variations of the N-position, some studies specify that the feet should be close together or shoulder width apart; while some describe subjects looking forward, others specify using a visual mark or point at eye level. An alternative variation of the N-position, referred to as the Toe-position in this article, was used in studies by Fan et al. [52] and Forsman et al. [53], where subjects returned to the N-position from a toe stand.

**Table 1:** Reference posture used to record a neutral trunk posture, indicating 0° trunk inclination or flexion. All studies were conducted in real work environments, and the selection was based on those identified by Lind et al. [27].

Reference Posture	
**Standing upright**	**Reference posture not reported** ^1^
[13,40,54,55,56,57,58,59,60,61,62,63,64,65,66,67,68,69,70,71,72,73,74,75,76,77,78,79,80,81,82,83,84,85,86,87,88,89]	[46,90,91,92,93,94,95,96,97,98,99,100,101,102]

Note: ^1^ not reported or information cannot be found.

The N-position appears to be the dominant posture for recording the reference zero-angle position for the trunk posture. It is also used to establish the reference zero-angle position for the upper arm, alongside other reference procedures, as summarized in Table 2. While the N-position does not involve lateral trunk inclination or typically require the use of an external dumbbell, most other reference procedures include these elements, typically using a 2 kg dumbbell. One of these procedures, referred to here as the “Stockholm procedure”, involves lateral trunk inclination and an external weight and is performed either standing or seated [44,89,103,104,105].

**Table 2:** Reference posture used to record a neutral arm posture, indicating 0° arm elevation. All studies were conducted in real work environments, and the selection was based on those identified by Lind et al. [27].

Lateral Trunk Inclination				No Lateral Trunk Inclination					Posture Not Reported
Standing		Seated		Standing			Seated		
**weight**	**no weight**	**weight**	**no weight**	**weight**	**no weight**	**not reported**	**weight**	**no weight**	
[89]	[106]	[52,53,54,55,56,57,64,67,74,83,88,91,104,106,107,108,109,110,111,112,113,114,115,116,117,118]	-	[119]	[14,75,79,120]	[65,70,71,73,76,77,78,87,121,122]	[103,123,124,125]	-	[46,80,84,86,93,95,97,98,99,101,102,126,127,128,129,130,131,132] ^1^

Note: ^1^ a weight was used.

However, the peer-reviewed literature provides limited information on the reproducibility and accuracy of these reference postures and similar approaches for measuring neutral trunk- and arm postures. Additionally, alternative procedures may offer improved reliability. Aarås and Stranden [48] evaluated the reliability of the N-position reference posture for the head and arms in five participants, reporting an average deviation from the midpoint of less than 1° for the head posture, 1.2° for arm flexion (i.e., arm elevation in the sagittal plane, forward), and 0.9° for arm abduction (i.e., arm elevation in the frontal plane, sideways). In a more recent study [69], median group differences before and after measurements were 3.8° for trunk sagittal inclination and 1.8° for trunk lateral inclination (Nastaran, Raffler. 2020. e-mail message to author, August 25). In our research, we have observed that this posture can occasionally be challenging to consistently adopt without postural divergence. While sensor-based inclinometers provide highly accurate measurements, calibration-related errors of approximately 4° in the reference posture can be unacceptably large, potentially obscuring actual improvements following an intervention [133]. To our knowledge, no study in the ergonomics literature has systematically evaluated the reliability of this reference posture for trunk angles on a larger sample or the reliability of alternative procedures.

With regard to the arm posture, Dahlqvist et al. [106,134] observed considerable differences between reference positions involving lateral (sideways) trunk inclination when a seated posture with an external weight was compared to standing reference positions without an external weight. For example, Dahlqvist et al. [134] reported group root mean square differences of 8.5° and 14° for the left and right arms, respectively. Furthermore, Jackson et al. [105] evaluated the accuracy of upper arm inclinometry against visually projected lines between 30° and 150°, with incremental steps of 30°. The inclinometers (triaxial accelerometers) were taped to the arm and a seated reference position with lateral trunk inclination with an external weight was used as the reference position (0° arm elevation). They reported that the inclinometers systematically underestimated the arm elevation angle, for example, by 13.5° and 11.1° on average at 90° arm abduction and arm flexion, respectively. The divergences were potentially attributed (at least partly) to soft tissue artifacts [105,135,136], and the authors presented conversion equations to allow for correction for the bias. Notably, this bias was not observed by Hansson [137] who, in a letter to the editor, reported an average divergence of ≤1° for the right and left arms when the participants held their arms at what was judged to be 90°.

### 1.3. Summary of Identified Research Gap

The identified literature highlights a lack of research on the reliability of reference postures for arm and trunk measurements. Additionally, it remains unclear whether the bias in arm elevation measurements reported by Jackson et al. [105] persists when motion capture devices are placed in the pockets of workwear, allowing for slight movement, rather than being taped directly to the arm.

### 1.4. Research Objective

Given these research gaps, the aim of this study was to evaluate the reliability of three reference positions for sagittal (forward/backward) trunk inclination and the accuracy of two reference positions for arm inclinometry using motion capture devices embedded in workwear.

## 2. Materials and Methods

### 2.1. Subjects

The study population (Table 3) comprised 22 women and 18 men, recruited from a working population pool at three organizations located in the Stockholm Region, Sweden. To ensure a broad working-age sample, the inclusion criteria required participants to be 18 years or older, currently employed or enrolled as students, and free from physical conditions, such as pain, disorders, or impairments that could hinder the execution of the reference procedures. Thirty-seven participants reported right-hand dominance and three reported left-hand dominance. Twenty participants had a BMI ≥ 23.0, and eleven had a BMI ≥ 25.0. There were no statistically significant differences in age or stature between these groups and the rest of the population. The study was approved by the Regional Ethics Committee in Stockholm, Sweden (2017/1586-31/4 and 2019-01206), and all participants provided written informed consent prior to participation.

### 2.2. Instrumentation

**Figure 1:**
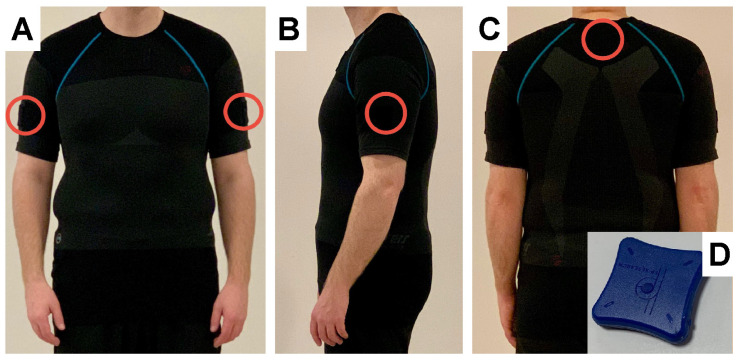
Positions of the inertial measurement units (IMUs) (here highlighted with red circles) from the anterior view (**A**), the lateral view (**B**), and the posterior view (**C**). The motion tracking device (i.e., the IMU) used (**D**).

Postural data for the trunk and upper arms were recorded using the Smart Workwear System [37,38]. The system included a stretchy workwear shirt (Model 9418, LiteWork, Snickers Workwear, Hultafors Group Sverige AB, Bollebygd, Sweden; fabric: 58% polyamid and 42% polyester 37.5^®^; mass: 165 g/m^2^) customized into a short-sleeved shirt with embedded pockets on the trunk and arms for IMUs (LPMS-B2, LP Research, Tokyo, Japan; dimensions: 39 × 39 × 8 mm; mass: 12 g). Participants were individually fitted with appropriately sized shirts (XS–L) to accommodate their body dimensions. To measure the trunk inclination, a single IMU was placed in the trunk pocket positioned on the upper back at the level of thoracic vertebrae 1–2 [45,52] (Figure 1). To measure the right upper arm elevation angle, a single IMU was placed in a pocket of the workwear enabling the upper edge of the IMU to be positioned approximately distal to the medial deltoideus muscle insertion [39,40,138] (Figure 1). The IMUs (Figure 1D) transmitted real-time data via Bluetooth to an Android 8.0 smartphone (Galaxy A5 2017, Samsung, Seoul, Republic of Korea), and the data were analyzed using the ErgoRiskLogger application, as described elsewhere [38]. Since the study focused on static postures, the IMU sampling rate was set to 25 Hz. The 3-axis accelerometer in the IMUs was set to a range, per axis, of ±4 G, and the 3-axis gyroscope was set to ±500°/s. A 16-bit resolution was used, followed by an extensive complementary Kalman filter (that is, a sensor fusion filter) to obtain the orientation of the sensor. As this study concerned static postures, it mainly used the accelerometer signals. According to the specification of the IMU, the static angle error was below 0.5°. More information on the Smart Workwear System is reported elsewhere [38].

### 2.3. Experimental Protocol

**Figure 2:**
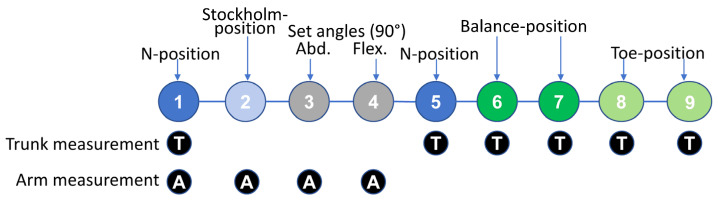
The order of the reference procedures and set angle trials.

**Figure 3:**
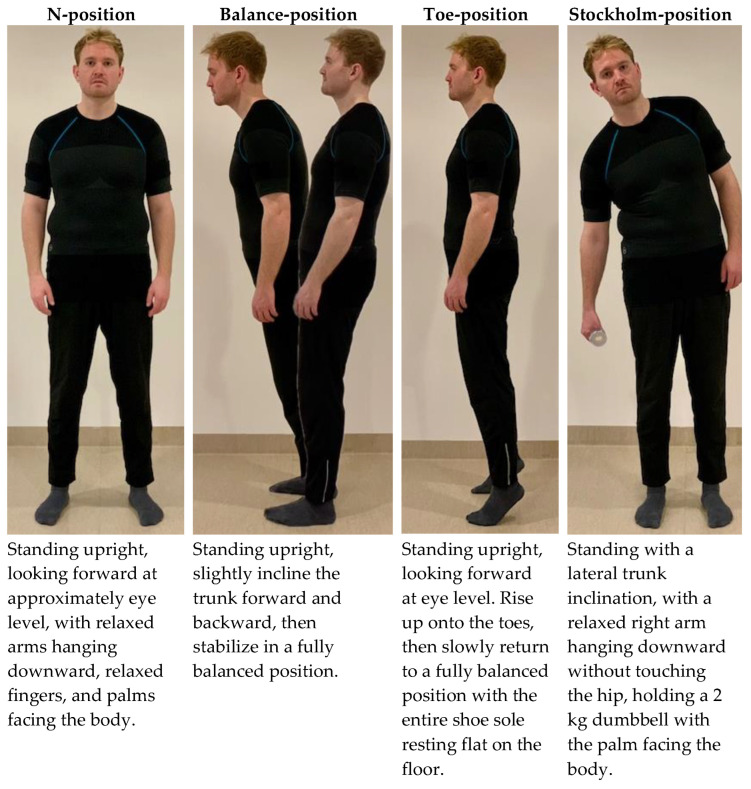
Description of the four reference procedures.

Four different reference positions were performed in a fixed order (Figure 2 and Figure 3). Trunk postures were assessed using three of the four positions, and arm postures were assessed using two positions. As shown in Figure 2, the evaluation began with recordings of the N-position, followed by the Stockholm-position. Subsequently, static arm elevation angles were recorded at a set angle of 90°. The N-position was then repeated. Finally, the Balance-position was repeated twice, and the session concluded with two repetitions of the Toe-Position.

The set angle procedure involved holding the upper arm still for 5 s in a 90° arm elevation (abduction), followed by another 90° arm elevation (flexion), with assistance from an instructor. The set angles were performed with a straight arm, maintaining a straight elbow and wrist, with the palm facing downward. The instructor (one of two physiotherapists) aligned the participants’ right arm by positioning the midline of the humerus parallel to the targeted abduction and inclination angles guided by the set angles printed on a large paper sheet (100 × 135 cm). This approach was modeled after the procedure described by Jackson et al. [105], but instead of visual projection, printed lines were used to eliminate the spatial distortion observed during preliminary pilot tests. Consistent with Jackson et al. [105], this assisted positioning was assumed to represent the “true” angle of the upper arm.

To record the postures of the trunk and arm in the version of the ErgoRiskLogger application we used, a global (original) reference recording was first made (the first N-position shown in Figure 2), and the other recordings were derived from this as a base. For arm postures, the global (original) value for the Stockholm-position was derived by subtracting the angular elevation difference (delta) between the global reference recording (i.e., 1st N-position) and the Stockholm-position. To illustrate this, if an elevation angle of 15° was recorded for the Stockholm-position, this value was equal to the difference compared to the global reference recording (i.e., 1st N-position). In the same way, the measured elevation at the set angle of 90° arm abduction and 90° arm flexion for the Stockholm-position was estimated as the individually measured angle in the global reference recording, with the addition of the individual delta.

### 2.4. Data Analysis

The data were checked for normality using the Shapiro–Wilk test (α = 0.05) and by visually inspecting histograms. For non-normally distributed data, the Wilcoxon signed-rank test was applied, while the paired samples t-test was used for normally distributed data.

For the trunk posture, the average trunk sagittal inclination angle was compared pairwise among the reference procedures using the Wilcoxon signed-rank test, as one of the three pairs was non-normally distributed.

The test–retest reliability was evaluated through pairwise comparisons among the three reference procedures using the Wilcoxon signed-rank test, as all three pairs were non-normally distributed.

For the arm posture, the differences in the arm elevation between the N-position and the Stockholm-position were compared pairwise at the set angles of 90° abduction and 90° flexion using the paired samples *t*-test.

Statistical analyses were performed using MATLAB R2023b Update 4 (The MathWorks, Inc., Natick, MA, USA), while normality tests were conducted using R (The R Foundation, version 2024.09.0). The significance level was set at α = 0.01.

## 3. Results

The results are based on 240 recordings of trunk postures and 160 recordings of arm postures, encompassing all 40 participants with no missing data.

### 3.1. Trunk Posture

The group mean differences in trunk inclination angles in the sagittal plane relative to the N-position are shown in Table 4. At the group level, the Balance-position resulted in a slightly more forward trunk inclination angle relative to the N-position (0.44°), while the Toe-position resulted in a slightly more backward trunk inclination angle (−0.33°); however, these differences were not statistically significant (*p* > 0.01).

#### Trunk Posture—Reliability

The test–retest reliability of the reference postures for trunk angles in the sagittal plane is presented in Table 5, based on data from 40 subjects, each performing two repetitions of each position (no missing values). The group mean absolute difference for the N-position and the Balance-position was 1.60° and 1.56°, respectively, with individual maximum differences exceeding 5°. The Toe-position demonstrated the lowest group mean absolute difference at 0.72°, i.e., the highest test–retest reliability, significantly higher than the two other reference postures (*p* < 0.005).

### 3.2. Arm Posture

#### Arm Posture—Accuracy

**Figure 4:**
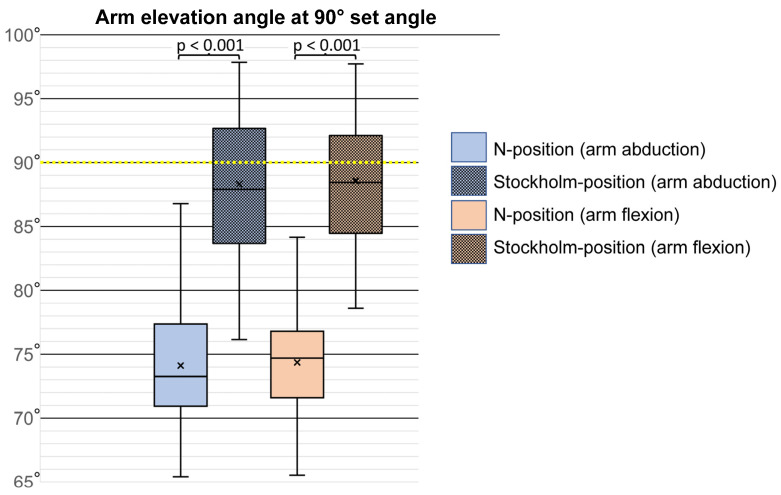
Box plots of arm elevation angles (abduction and flexion) at 90° set angles (yellow dotted line) for the two reference positions: N-position and Stockholm-position. The box represents the interquartile range (25th to 75th percentiles) and the median, while the mean is marked with an “x”. Vertical lines extending from the box indicate adjacent values, which are the most extreme values within 1.5 times the interquartile range for each group.

The corresponding values for the N-position and Stockholm-position at the 90° set angle for abduction and flexion are displayed in Figure 4 and Appendix A.

For arm abduction, using the N-position as a reference resulted in an average elevation angle of 74.1° (SD 4.7), which is 15.9° lower than the assumed true angle. In comparison, the Stockholm-position produced an average elevation angle of 88.3° (SD of 5.5), which was 1.7° lower than the assumed true angle.

For arm flexion, the N-position yielded an average elevation angle of 74.4° (SD of 3.9), which was 15.6° lower than the assumed true angle. In comparison, the Stockholm-position resulted in an average elevation angle of 88.6° (SD of 4.7), i.e., 1.4° lower than the assumed true angle. Hence, the Stockholm-position consistently yielded values that were substantially closer to the assumed true angle, and the angular differences between the N-position and the Stockholm-position at the 90° set angle were statistically significant (paired samples t-test) for both arm abduction and arm flexion (*p* < 0.001).

## 4. Discussion

### 4.1. General Summary of the Findings

This study, which included 40 subjects, evaluated commonly used reference procedures for obtaining the reference zero-angle positions for measurements of trunk and arm postures. The findings support the conclusion that the choice of reference procedure is critical, as it can significantly affect the reliability and accuracy of technical measurements using sensor-based inclinometers.

For recordings of trunk postures, the results of this study suggest that the test–retest reliability can be improved by replacing the commonly used reference posture (referred to as the N-position) with an alternative reference procedure (referred to as the Toe-position) where the participants arrive at a balanced upright posture from a toe stance.

For recordings of arm postures, the results show that the commonly used N-position, performed in an upright posture without lateral inclination, can lead to substantial and systematic underestimation of arm elevation angles relative to the reference, with errors of approximately 15° for both abduction and flexion if the bias is not corrected. In contrast, the use of an alternative reference procedure (position), i.e., the upright Stockholm-position, which involves lateral trunk inclination and the use of an external weight, resulted in group mean values that closely matched the reference angles, with deviations of less than 2°. These findings highlight the importance of incorporating lateral trunk inclination, and possibly an external weight, to improve measurement accuracy.

### 4.2. General Interpretation of the Results and Comparisons with Related Literature

This study contributes to the identified research gap by providing new information on the reliability of a commonly used procedure for achieving accurate recordings of a neutral trunk posture, based on a relatively large and diverse sample of men and women spanning various age groups.

#### 4.2.1. Trunk Sagittal Inclination

Regarding trunk postures, the group mean (absolute) test–retest differences in the trunk sagittal inclination in the current study were smaller for all three positions evaluated (i.e., all ≤ 1.60°) compared to the median group differences of 3.8° in the study by Raffler et al. [69]. Notably, Raffler et al. [69] measured the postures before and after the measurement of work activities, which lasted about 1–2 h. One potential reason for the difference could be related to changes in the position of the motion device, which can occur, especially during prolonged use, dynamic activities, or when motion devices are insufficiently secured. The group mean (absolute) test–retest differences in the trunk sagittal inclination in the current study, especially for the Toe-position (0.72°), were close to the test–retest values for the neck (head) of 0.85° reported by Aarås and Stranden [48].

#### 4.2.2. Arm Elevation

Concerning arm postures, contrary to findings from earlier studies by Bernmark and Wiktorin [44], Jackson et al. [105], Lind and Forsman [139], and the related study by Lind et al. [140], this study did not confirm a considerable bias in arm posture recordings at a 90° arm elevation. Instead, and in agreement with what was reported by Hansson [137], who used a seated reference posture, the observed values in the current study closely approximated the assumed true angle, with only slightly lower values of <2°. This can be compared with the difference from the assumed true angle noted by Jackson et al. [105], which averaged 13.5° and 11.1° at a 90° arm abduction and arm flexion, respectively. Previous research has developed equations to harmonize data across different instruments and calculations [52,53,141] or to correct inaccuracies potentially caused by soft tissue artifacts and reference procedures [105]. The large underestimation of arm elevation angles (approximately 15°) is likely caused by the arm, although relaxed, being more abducted when leaning against the body.

### 4.3. Strengths and Limitations

It should be noted that this study is limited to the sagittal trunk inclination and arm elevation. Although these are the most common exposure metrics for postures of the trunk and upper arm in the ergonomics literature related to WMSD outcomes [27], future studies are needed to explore the reference position in relation to, e.g., trunk sagittal inclination.

Regarding reference procedures for obtaining the reference zero-angle position for the trunk, while the study’s findings suggest that the reliability can be improved through alternative procedures, the accuracy of these reference positions was not directly evaluated. Furthermore, it is debated which posture truly represents the “natural” or “neutral” upright posture [49,50]. However, since high accuracy depends on precision, some conclusions can still be drawn regarding the potential advantages of adopting alternative procedures to enhance both the reliability and overall measurement accuracy. A potential limitation was that the reference procedures were not performed in a randomized order, which could introduce learning effects, particularly for reference positions performed later. However, all participants were already familiarized with the reference procedures from a prior trial [140], with the exception of the Balance-position and Toe-position. Therefore, it seems unlikely that the relatively higher test–retest reliability observed for the Toe-position was due to learning effects.

With respect to reference procedures for obtaining the reference zero-angle position for the arm, while the results provide important findings, the accuracy was evaluated only at a 90° arm elevation angle (flexion and abduction). Consequently, the bias may vary in magnitude at other angles. Furthermore, additional information is necessary to develop conversion equations for correcting bias, particularly for the N-position, which resulted in substantial bias. Although only the standing version of the Stockholm procedure was included in this study, preliminary findings from a related study suggest that the differences between the standing and seated variations are small [140].

Additionally, the global (original) reference recording for arm abduction and flexion for the Stockholm-position was derived by subtracting the angular elevation difference between the first N-position and the Stockholm-position (as described in Section 2.3). This approach was necessitated by limitations in the version of the Smart Workwear System used. Preferably, another global (original) reference recording should have been obtained when having the participants stand in the Stockholm-position. The present method introduces an approximation in determining the angle between the Stockholm-position and the 90° arm elevations. Nevertheless, this adjustment likely has a negligible effect on the abduction angle recorded at the 90° set angle, and a negligible-to-minimal effect on the flexion angle. Future studies are, however, recommended to further investigate this.

A strength of this study is the inclusion of a relatively large and diverse sample of subjects, encompassing both women and men across a wide age range (26–70 years). This improves the generalizability to other populations. The stature and body mass of the women in this study were slightly lower than those reported among a Swedish female working population [142] with a median stature of 167.3 cm and median body mass of 64 kg. This stature approximates that of US women aged 40–49 years (162.6 cm) but with a higher body mass (75.2 kg) [143]. The men’s stature and body mass were approximately equal to the median stature of 177.9 cm and body mass of 75 kg among a Swedish male working population [142]. This stature approximates that of US men aged 40–49 years (176.5 cm), who have a higher body mass (90.5 kg) [143].

All measurements were recorded with static (isometric) postures, without involving any movement. The potential improvement in the measurement accuracy that can be attributed to having the sensors embedded in tight workwear (as opposed to taped) may differ during tasks involving high movement velocities, especially if the fit of the workwear is loose in some parts where the sensors are embedded, as pointed out by Hoareau et al. [144].

### 4.4. Practical Implications and Future Research

The bias attributed to reference procedures seems to have been underestimated, especially as measurements from technical measurement instruments such as IMUs and accelerometers are often regarded as having superior accuracy and precision when compared to self-reports and systematic observations [54,145,146,147,148].

The main implication of this study is that an upright neutral position is not recommended for recording the neutral position of the upper arm. Instead, the reference position should include lateral trunk inclination. As a result, findings from studies that assume that the N-position represents zero arm elevation (without additional corrective measures) may not be directly comparable to those that use other reference positions. Furthermore, it is questionable whether the N-position should be used at all for recording arm postures without corrections. Given the relatively common use of the N-position, as shown in Table 2, caution is warranted when interpreting the results of studies that did not account for the bias associated with the N-position as observed in the current study. This also underscores the need for conversion methods to convert data obtained from different reference procedures, particularly when one involves trunk inclination and the other does not.

While the current study demonstrated that a posture including trunk inclination and an external load can yield relatively accurate arm elevation angles (both abduction and flexion), the divergent results from the related study by Lind et al. [140], as well as findings from Jackson et al. [105], Bernmark and Wiktorin [44], and Lind and Forsman [139], underscore the need for further research to better understand the reasons behind these discrepancies. The results of the current study highlight the need for the conversion of postures obtained using different reference positions to ensure accuracy and comparability between studies.

To improve the test–retest reliability in recording a neutral trunk position, and consequently the accuracy of the N-position, future studies could explore the feasibility and efficiency of increasing the number of measurements, allowing for the calculation of an average value based on multiple recordings taken before and after the work activity, or alternatively, consider using the Toe-position.

## 5. Conclusions

The choice of reference procedures for obtaining reference zero-angle positions can significantly impact the reliability and accuracy of technical measurements using sensor-based inclinometers. For recordings of a zero-angle trunk position in the sagittal plane, the commonly used N-position (or N-posture, or I-pose) was observed to exhibit some imprecision. An alternative posture, where participants arrived at the N-position from a toe stand, improved the test–retest reliability.

For recordings of arm elevation, the N-position was compared with an alternative procedure (the Stockholm-position), which includes lateral trunk inclination. The N-position resulted in a substantial average bias of 15°, raising concerns about its use without adjustments to mitigate this error. In contrast, the Stockholm-position demonstrated higher accuracy, with deviations averaging less than 2°, making it the superior option of the two.

The findings of this study can guide the selection of appropriate reference procedures for both field and laboratory applications. Additionally, the findings highlight the importance of incorporating lateral trunk inclination when recording the reference zero-angle position of the arm.

## Figures and Tables

**Table 3:** Summary of participant characteristics (SD, standard deviation).

Demographic Variables	Women, Mean (SD)	Men, Mean (SD)
N	22	18
Age (years)	42.0 (13.4)	42.5 (10.7)
Body mass (kg)	61.3 (9.7)	76.8 (10.6)
Stature (cm)	164.0 (6.5)	178.7 (8.4)
BMI (kg/m^2^)	22.7 (2.8)	24.0 (2.2)

**Table 4:** Group mean differences in trunk angles in the sagittal plane (forward/backward) relative to the N-position and the statistical differences (Wilcoxon signed-rank test) of the group differences.

	Angular Differences		Statistical Differences		
**N-position**	**Balance-position**	**Toe-position**	**N-position vs.** **Balance-position**	**N-position vs.** **Toe-position**	**Balance-position vs.** **Toe-position**
Ref.	0.44°	−0.33°	*p* = 0.095	*p* = 0.622	*p* = 0.067

Notes: Angular differences with positive values indicate a forward sagittal trunk inclination relative to the reference, and negative values indicate a backward sagittal trunk inclination relative to the reference.

**Table 5:** Average absolute test–retest differences in the sagittal trunk inclination (forward/backward) of the reference positions to record a neutral trunk angle, first versus second trial, expressed as the mean (standard deviation, SD), and the statistical differences (Wilcoxon signed-rank test) of the group mean differences. The results are based on 40 subjects, each performing two repetitions of each reference position.

Absolute Group Differences (SD) for 1st vs. 2nd Recording			Statistical Differences		
**N-position**	**Balance-position**	**Toe-position**	**N-position vs.** **Balance-position**	**N-position vs.** **Toe-position**	**Balance-position vs.** **Toe-position**
1.60° (1.37°)	1.56° (1.29°)	0.72° (0.54°)	*p* = 0.717	*p* < 0.005	*p* < 0.001

## Data Availability

The raw data supporting the conclusions of this article will be made available by the authors on request.

## Appendix A

**Table A1:** Mean (SD) arm elevation angles (abduction and flexion) at 90° set angles for two reference positions: N-position and Stockholm-position.

Arm Abduction		Arm Flexion	
**N-position**	**Stockholm-position**	**N-position**	**Stockholm-position**
74.1 (4.7)	88.3 (5.5)	74.4 (3.9)	88.6 (4.7)
